# Neuroanatomical Variability of Religiosity

**DOI:** 10.1371/journal.pone.0007180

**Published:** 2009-09-28

**Authors:** Dimitrios Kapogiannis, Aron K. Barbey, Michael Su, Frank Krueger, Jordan Grafman

**Affiliations:** 1 Clinical Research Branch, National Institute on Aging (NIA), National Institutes of Health (NIH), Baltimore, Maryland, United States of America; 2 Cognitive Neuroscience Section, National Institute of Neurological Disorders and Stroke (NINDS), National Institutes of Health (NIH), Bethesda, Maryland, United States of America; 3 Department of Psychology, Georgetown University, Washington, D. C., United States of America; Victoria University of Wellington, New Zealand

## Abstract

We hypothesized that religiosity, a set of traits variably expressed in the population, is modulated by neuroanatomical variability. We tested this idea by determining whether aspects of religiosity were predicted by variability in regional cortical volume. We performed structural magnetic resonance imaging of the brain in 40 healthy adult participants who reported different degrees and patterns of religiosity on a survey. We identified four Principal Components of religiosity by Factor Analysis of the survey items and associated them with regional cortical volumes measured by voxel-based morphometry. Experiencing an intimate relationship with God and engaging in religious behavior was associated with increased volume of R middle temporal cortex, BA 21. Experiencing fear of God was associated with decreased volume of L precuneus and L orbitofrontal cortex BA 11. A cluster of traits related with pragmatism and doubting God's existence was associated with increased volume of the R precuneus. Variability in religiosity of upbringing was not associated with variability in cortical volume of any region. Therefore, key aspects of religiosity are associated with cortical volume differences. This conclusion complements our prior functional neuroimaging findings in elucidating the proximate causes of religion in the brain.

## Introduction

Religious behavior is a uniquely human phenomenon without accepted animal equivalents [1], [2], present in all modern cultures and evident in archaeology from all periods of human history and pre-history [2], [3]. For an explanation of religion, we turn to Tinbergen's distinction between ultimate (or evolutionary) and proximate (causal and developmental) explanations [4]. Much of the scientific literature on religion attempts to create ad hoc explanations and explain the observed range of religious phenomena, in light of evolutionary benefits at the personal and community levels [1], [2]. Other models *draw analogies* between the religion phenotype and that of adaptive animal behaviors [1], [2], [5] or *simulate* religious behavior de nova, using pre-specified parameters [2], [6]. While these approaches bring certain benefits, they nevertheless lack a clear understanding of the proximate neural circuitry that supports religious beliefs. Cognitive Neuroscience attempts to create an explanatory schema that includes both proximate and ultimate causes, by linking the emergence of religion in our ancestors with the development of novel cognitive processes, such as Theory of Mind (ToM) [7], [8], social cognition [1], [7], [8] and symbolic language [3], which, in turn, have different evolutionary origins [1] and presumably resulted from expansion of specific brain regions (such as the prefrontal cortex, PFC, the precuneus, the temporal lobe, etc) [7]. (In this article, we use the term “religion” or “religious behavior” to designate shared *beliefs, practices and experiences* regarding supernatural agents. We use the term “religiosity” to designate a set of psychological and behavioral traits related to adoption of religious beliefs and engagement in religious behavior. We use the term “God” to refer to the target of shared religious commitment in Western traditions. For simplicity, and accuracy, we use the pronoun “He” to denote this target, because God is typically described in these traditions as personal, gendered, and male.)

In order to discover the proximate causes of religion in the brain, our group initially focused on religious *beliefs* and their cognitive architecture. We found that religious beliefs (at least in the North American Western traditions we studied) are organized cognitively along three dimensions, which constitute a conceptual space. The first two reflect perceptions of 1) Gods' involvement and 2) God's emotion. A third reflects judgment over 3) the source of religious knowledge ranging from doctrinal to experiential [9]. The neural correlates of these dimensions were found to be brain networks which had been previously implicated in understanding agents' actions and intent-related ToM (for Dimension 1), emotion-related ToM and emotional regulation (for Dimension 2) and abstract semantic processing and imagery (for Dimension 3) [9]. By differentially engaging these networks, individuals construct religious belief representations, which are subsequently adopted or rejected based upon cognitive-emotional interactions within the anterior insulae [9]. Religious belief systems presumably interact with other belief systems, social values [10] and morals, and help determine long-term goal selection, behavioral control and emotional balance [11].

Other functional neuroimaging studies have sought to discover the neural correlates of various religious acts or *practices*, which *presumably* recreate religious *experiences* inside the scanner. In these studies, subjects have performed (among other tasks) formalized and improvised praying [12], [13], “mystical” praying [14] and meditation [15]. These studies have reported several functional brain correlates to these practices, with variable engagement of both subcortical [12] and cortical areas which had been previously implicated in social cognition [13]. Their findings have not yet been integrated in any comprehensive neural-cognitive model for religious *experience*. Nevertheless, the variability of their results is informative by itself, since it seemingly rules out that any single area is modularly or modally specific to religious experience (a so-called “God-spot”).

A strategy, for discovering the proximate causes of complex cognitive functions, which, in turn, may inform novel evolutionary explanations, is to associate variability at the level of the behavioral phenotype and (structural or functional) variability at the level of the likely proximate causes (such as at the levels of genetics and/or the brain). For instance, such an approach has been used to discover the proximate causes of emotional and non-emotional memory [16], [17], [18], [19] and face recognition [20], [21]. Religion seems suitable for such an approach, since religiosity varies widely among modern humans, a fact attributable to environmental and genetic factors [22], [23] and to its interaction with other personality and social behavior traits [11], [24], [25]. In this study, we hypothesized that religiosity is tied to neuroanatomical variability and tested this idea by determining whether components of religiosity were predicted by variability in regional cortical volume measured by magnetic resonance imaging (MRI).

Forty healthy participants with varying patterns of religiosity participated in this and an already published parallel functional MRI (fMRI) study [9]. The purpose of the fMRI study was to identify cognitive processes and brain networks engaged by exposure to a range of religious beliefs. In the VBM study reported below, on the other hand, we assumed that religiosity patterns are based on clusters of personality traits that influence cognitive strategies and behavior over time. For measurement of these traits, we relied on participants' self-reporting about their current religious experience and behavior, their religious upbringing, and about aspects of their worldview (See Supporting Table S1). All survey items were presented as 7-point visual analog Likert scales and subjects had to report the degree to which they currently experience (or had experienced during their upbringing) the content of the item. These items were subsequently entered in a Principal Component (PC) Factor Analysis (FA) to identify common themes underlying their responses. Then, to identify regions of gray matter whose volume is associated with these traits, a VBM analysis of gray matter was performed with the identified principal components as regressors.

## Results

### Factor Analysis (Table 1)

**Table 1:** The rotated correlation matrix of the original survey items and the four Principal Components (PCs).

	Principal Components			
Survey Item	PC1	PC2	PC3	PC4
Degree of current religiosity	.906			
Degree of current religious participation	.917			
Current frequency of praying	.918			
Current frequency of praying in private	.830			
Current frequency of reading of Scripture	.852			
Degree to which religion influences important decisions	.803		.347	
Belief in Life after Death	.778			.427
Belief in Heaven	.798			.418
Belief in Hell	.792		.312	.308
Belief in a personal God	.765			.337
Perception of God’s Love	.875			
Praying for forgiveness of sins	.866			
Fear of God’s anger				.911
Seeking God’s will	.939			
Perception of God’s proximity	.911			
Perception of God’s awareness	.917			
Perception of God’s friendship	.911			
Perception of God’s fellowship	.941			
Relying of God for important decisions	.915			
Doubting God’s existence	−.617		−.561	−.318
Religiosity during upbringing		.829		
Religious participation during upbringing		.881		
Praying during upbringing		.899		
Endorsement of “Eat, drink and be merry, because tomorrow we die”			−.816	
Belief that humanity is equally good and bad			−.829	
Belief that values are relative depending on the situation		−.604	−.385	
Belief that life has an ultimate purpose	.776			

Four Principal Components (PCs) were identified; they can be best explained by those survey items with the highest and most exclusive loadings. Items suggesting both an intimate relationship with God (such as experiencing God's fellowship and seeking his will) and engagement in religious behavior (such as prayer and religious participation) loaded on the 1^st^ PC (PC1). Items referring to religiosity of upbringing loaded positively and an item implying moral relativism loaded negatively on the 2^nd^ PC (PC2). Doubting God's existence, endorsement of “Eat, drink and be merry, because tomorrow we die” [a saying epitomizing the Epicurean and Hedonistic philosophic traditions, which advocated a worldview contrasting sharply with the one advocated by the Judeo-Christian religious tradition], and considering humanity as equally good and bad, loaded negatively on the 3^rd^ PC (PC3). Therefore, PC3 can be best understood by these negative correlations with elements of non-religious pragmatism. Finally, fear of God's anger loaded on the 4^th^ PC (PC4).

### VBM (Table 2)

**Table 2:** Cortical areas whose volume is associated with the PCs.

Regressor	Voxel coordinates	Z value	Cluster size	t-statistics significance	Brodmann area	Localization
**PC1: Experiencing an intimate relationship with God (positive correlation)**	(58, 6, −20)	4.01	382	Cluster level correction (p = 0.030)	BA 21	R middle temporal gyrus
	(54, 12, −26)	3.85	Above cluster	Cluster level correction (p = 0.030)	BA 21	R temporal pole
**PC2: Religiosity of upbringing**	No areas exceeded threshold					
**-PC3: Non-religious pragmatism (positive correlation)**	(14, −68, 32)	4.58	574	Cluster level correction (p = 0.005)	BA 7	R precuneus
				FWE correction (p = 0.075)		
	(20, −56, 20)	3.79	Above cluster	Cluster level correction (p = 0.005	BA 17	R calcarine gyrus
**PC4: Experiencing fear of God’s anger (negative correlation)**	(−10, −66, 52)	4.18	464	Cluster level correction (p = 0.014)	BA 7	L precuneus
	(−32, 62, −10)	3.87	351	Cluster level correction (p = 0.041)	BA 11	L orbitofrontal cortex

#### PC1

**Figure 1:**
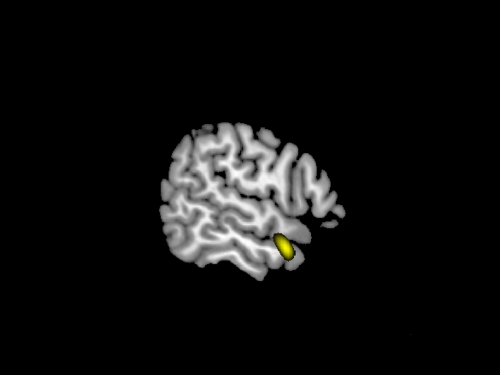
Experiencing an intimate relationship with God (PC1) positively correlated with cortical volume at the R middle temporal gyrus (MTG), BA 21, extending to the temporal pole. Threshold was set to p<0.001 uncorrected for visualization.

Experiencing an intimate relationship with God positively correlated with cortical volume of BA 21 at the R middle temporal gyrus (MTG) and its extension at the temporal pole (Fig. 1).

#### PC2

There were no cortical areas whose volume correlated with religiosity of upbringing.

#### - PC3

**Figure 2:**
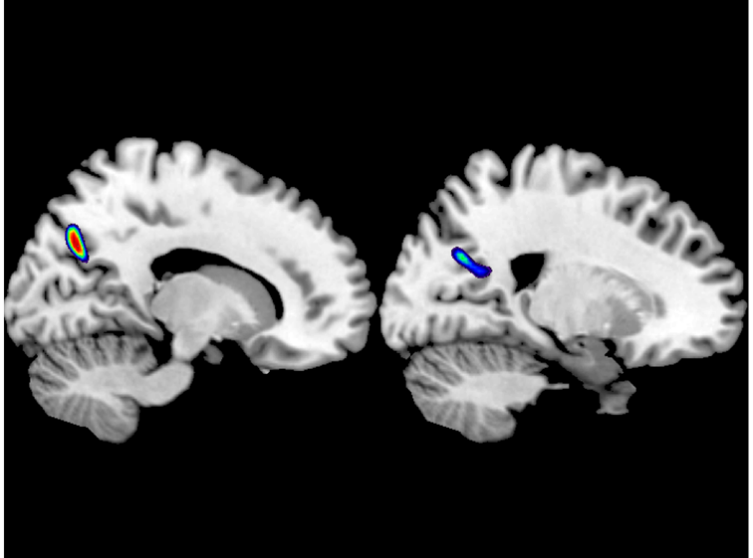
Non-religious pragmatism (the inverse of PC3) positively correlated with cortical volume at the R precuneus, BA 7 and the R calcarine gyrus, BA 17. Threshold was set to p<0.001 uncorrected for visualization.

Non-religious pragmatism (the inverse of PC3) positively correlated with cortical volume at the R precuneus, BA 7 and the R calcarine gyrus, BA 17 (Fig. 2). (Alternatively stated, PC3 negatively correlated with cortical volume at the R precuneus, BA 7 and the R calcarine gyrus, BA 17).

#### PC4

**Figure 3:**
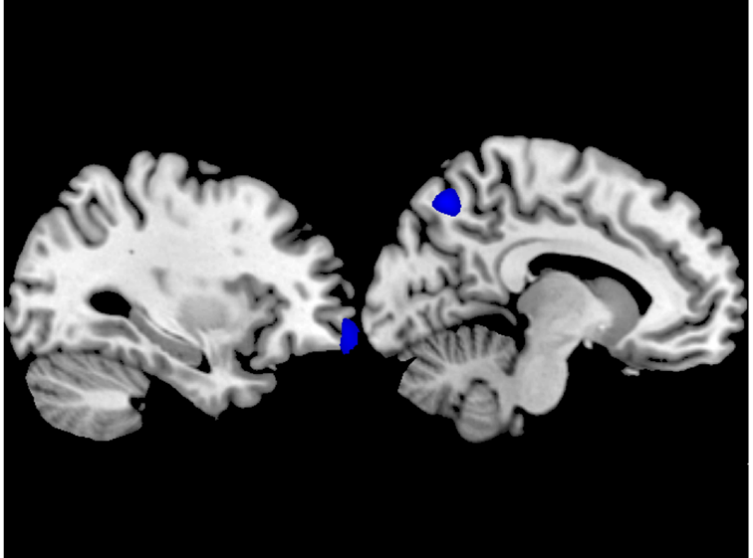
Experiencing fear of God's anger negatively correlated with cortical volume at the L precuneus, BA 7 and the L orbitofrontal cortex, BA 11. Threshold was set to p<0.001 uncorrected for visualization.

Experiencing fear of God's anger negatively correlated with cortical volume at the L precuneus, BA 7 and the L orbitofrontal cortex, BA 11. In other words, increased cortical volume in these areas predicted non-threatening God-related beliefs (Fig. 3).

## Discussion

In this study we identified structural brain variability associated with aspects of long-term *religiosity*. By defining brain regions that are tied to religion-related psychological and behavioral traits, we complemented our prior fMRI findings of networks involved in navigating the conceptual space of religious *beliefs* (and adopting those beliefs). The two methods assessed different levels of brain organization (functional recruitment during task performance vs. structural changes resulting from brain development and plasticity). Combined, their findings advance our understanding of the proximate causes of religion in the brain.

Relationships between regional cortical thickness or volume (measured with an unbiased and automated data analysis system) and cognitive task performance have been validated for a range of tasks [26]. Here, we show that such relationships exist in relation to the traits represented by the PCs of religiosity. These relationships, though, need not be considered as simple linear ones (i.e. increased brain volume resulting from/leading to trait manifestation), similarly to linear relationships demonstrable for simple tasks (i.e. increased brain volume resulting from/leading to improved task performance) [26], [27]. Both religious belief and religious practice functionally engage areas which are more broadly involved in social cognitive processing [9], [13]. Such areas are not selectively engaged by specific tasks or tied to unique brain functions [28], unlike the highly specialized motor and visuospatial areas or the hippocampus, the cortical density of which has so far been shown to change with practice [29], [30], [31], [32]. (Changes captured by MRI presumably represent changes in synaptic density [33], myelination within the grey matter [34] and other histologic alterations.) Nevertheless, the effect of personality traits (such as religiosity) can be viewed as selectively engaging complex sets of cognitive processes and representations over time, in response to similar situational demands [35]. Such selective and repeated engagement of networks involved in social cognition [28] may also result in practice-related changes in them. Some evidence for this exists in relation to certain personality traits [36] and complex cognitive practices, such as meditation [37]. Moreover, any practice-induced cortical changes do not rule out the possibility of pre-existing innate regional cortical differences predisposing people to certain traits and behaviors [29]. (Processes involving the white matter, such as myelination and synaptogenesis, are known to underlie the expansion of cognitive functionality in humans, from developmental [34] as well as from evolutionary perspectives [38]. This study was not designed to assess any micro-structural white matter changes or their relationship to religiosity.)

In relation to the FA results, it is interesting to note that items referring to religious behavior (such as praying and religious participation) clustered in PC1 with items referring to an intimate relationship with God (such as experiencing God's fellowship), rather than in PC4 with items reflecting fear of God. Whether this results from a pattern of religion based on non-threatening God representations which has prevailed in modern western society or is a human universal is an open question.

PC1, both reflecting intimacy with God and predicting religious behavior, correlated with cortical volume of the R MTG, BA 21. The R temporal lobe has long been suggested as a locus for religion in the brain [39]. In our parallel fMRI study, R BA 20 and 21 were engaged by doctrinal religious knowledge referring to the most abstract attributes of God [9]. Moreover, the R MTG, BA 21 and R IFG, BA 45, were co-activated as part of a network which was thought to mediate ToM in regards to God's intent and emotion [40], [41] and relate these to one's self [9]. This co-activation may be explained by the fact that the MTG (and neighboring lateral temporal areas) is (are) strongly interconnected in humans with the inferior frontal gyrus (IFG) via the arcuate fasciculus; phylogenetically, this neural network is considered crucial for the emergence of uniquely human cognitive abilities (such as symbolic language [42], [43]). In particular, the R MTG is important for self-processing (underscored, for instance, by its depressed activity in depersonalization disorder) [44], monitoring the status of intimate relationships (such as a mother's with her own child) [45] and setting boundaries between representations of intimate others (such as one's own mother) and one's self [46]. Therefore, by evolution of this area and its connections, a *personal* relationship with God as an intimate other may have become *possible*, allowing modern humans to experience this bonding– which they variably do. There is evidence that the R MTG is indeed structurally variable in modern humans and this variability has important clinical implications: R MTG volume is decreased in adolescent and first-episode schizophrenia patients [47], [48] and increased in obsessive compulsive disorder (OCD) patients [49]. We speculate that the range of R MTG volumes can be viewed as a spectrum, in which high R MTG volume is associated with stereotyped and ritualistic behavior, high-normal volume is associated with religious behavior (which, we should note, is by definition ritualistic), low-normal volume is associated with non-religiosity, and pathologically low volume is associated with schizophrenia (in which disorganized behavior and aberrant religiosity, with blurred boundaries between the self and God, may occur).

Experiencing fear of God's anger (PC4) was negatively correlated with volume of BA 11 and L precuneus, BA 7. BA 11 consists of phylogenetically newer granular cortex, associated with our ability for emotion-related ToM (also termed cognitive empathy) [50]. This area also exerts an emotional regulatory effect [51], inhibiting excessive emotional responses to negative stimuli to prevent them from interfering with performance under regular conditions [52], being itself inhibited in the face of grave danger [53]. In regards to the role of precuneus in religion, we have already proposed that it helps relate the representation of God to the self [9], [54] and, on that basis, we interpret its activation by personal praying in devout Christians [13] and the finding of the current study that increased volume of the L precuneus prevents fear of God (suggesting a enhanced ability to relate God to the self). The precuneus may also help provide context to the religious experience by retrieving memories and relating them to current situations [54], [55]. Therefore, people with lower cortical volumes in BAs 7 and 11 may be prone to a fear-based approach to God because of being compromised in representing the intentions and emotional disposition of God [50], regulating their emotions and modulating fearful responses towards a perceived powerful agent [56], as well as because of deficient engagement in personal (conversational) prayer with Him [13].

Moreover, an enhanced ability to switch between different perspectives to address moral dilemmas [54], [57] may explain why people with increased R precuneus volume tend to consider humanity as both good and bad and moral values relative (items loading on PC3, see Table 1). We speculate that people with increased R precuneate volume may also place an emphasis on worldly experiences over the inner life of imagination [58], which, in turn, may predispose them to adopt a non-religious life stance.

This study is correlational; therefore, it does not imply causality. Moreover, it was performed in adults. Subjects may have been predisposed to follow specific patterns of religious behavior by their individual brain development or their religious behavior may have contributed to volume changes of certain brain areas. Regardless, the fact that there is no brain area correlating with religiosity of upbringing (PC2) argues against religious nurture independently accounting for regional brain variability. Therefore, religiosity in adult life may reflect innate “susceptibilities”, perhaps genetic or *early* developmental, which are non-modifiable during upbringing, or any initial effect of religious upbringing may be dissipated by experiences in later life.

The brain areas identified in this and the parallel fMRI studies are not unique to processing religion, but play major roles in social cognition. This implies that religious beliefs and behavior emerged not as sui generis evolutionary adaptations, but as an extension (some would say “by product”) of social cognition and behavior. Furthermore, the current study suggests that evolution of certain areas that advanced understanding and empathy towards our fellow human beings (such as BA 7, 11 and 21) may, at the same time, have allowed for a relationship with a perceived supernatural agent (God) based on *intimacy* rather than *fear*. The idea that how you relate to “thy God” parallels how you behave to “thy neighbor” is a long-cherished claim of many religions (and is backed by some empirical evidence [11], [59]). In this study, we see that this link is elaborated in the cognitive and neural foundations of religion.

## Methods

The Neuroscience Institutional Review Board of the National Institutes of Health has approved this research. Informed written consent has been obtained and all clinical investigation has been conducted according to the principles expressed in the Declaration of Helsinki.

Healthy right-handed healthy adults were recruited through posting of the study on the website of the National Institutes of Health and word of mouth. Subjects provided medical history and underwent a screening neurological exam with a neurologist. Subjects with neurological or psychiatric disease or not able to undergo MRI were excluded from participation. In particular, we exclusion subjects with psychiatric diseases or first degree relatives of psychiatric patients, as well as of those with deleterious habits, such as alcoholism or substance abuse. No requirement for participation was made in regards to religiosity. Forty eligible subjects (20 women and 20 men; mean age = 35.7; mean years of education = 17.5) were, then, subjected to structural (as well as functional [9]) brain MRI and completed a survey on their religiosity.

The correlation matrix of survey items was subjected to Principal Components (PC) Factor Analysis using SPSS 17.0. Factors were extracted based on Eigenvalue <1. A varimax rotation with Kaiser normalization was applied to the solution to minimize the number of variables that have high loadings on each PC and to simplify the interpretation of the PC. Anderson-Rubin factor scores for the PCs were calculated for each subject. These scores are produced so that they have a mean of 0, a standard deviation of 1 and are uncorrelated.

A 3T GE MRI scanner (GE Medical Systems, Milwaukee, WI) and an 8-channel head coil were used to acquire high-resolution T1-weighted 3-dimensional magnetization-prepared rapid gradient-echo structural images. We used the unified segmentation algorithm [60], [61] implemented on SPM5 (Wellcome Department of Imaging Neuroscience, Institute of Neurology, UCL) to acquire images of gray matter, white matter and cerebrospinal fluid. We then performed modulated normalization of the gray matter images and smoothing with a 8-mm full-width at half-maximum filter. The resulting images were entered in a multiple regression analysis model, with the factor scores for the four PCs as covariates of interest. Moreover, age, gender, education and total intracranial volume were included in the model as covariates of no-interest, since we wanted to control for their effect on regional grey matter volume. In particular, age was an important confounder and, therefore, variance associated with it needed to be accounted for, since it is known to affect cognitive performance across a range of tasks, cortical grey density and volume throughout life [34], [62], [63], [64], [65], [66], and may also be related with changes in religiosity. Design orthogonality was calculated in SPM5 and no collinearity among the PCs or among the PCs and the other covariates was observed. Global effects for gray matter were calculated and global normalization with Analysis of Covariance (ANCOVA) was performed to localize regions where the trends in GM volume differ from global GM effects [67]. The effect of each covariate of interest was individually assessed at the whole brain level. The uncorrected statistical threshold for voxels at the whole brain level was set to p<0.001, with a minimum cluster size of 300, and, for detected clusters, cluster-level correction was implemented. Non-Stationary Cluster Extent Correction, which corrects for non-isotropic smoothness of VBM data [68] was also applied.

This study is correlational, bridging distant levels of biological organization (i.e. the cellular and tissue level, to the degree that its features are captured by MRI, and that of long-term behavior). The observed effects could be mediated by behavioral confounders influencing intermediate levels of organization (altering, for instance, patterns of self-care or habits, such as alcohol drinking). Such intermediate levels include the levels of the organ (brain), the organ system (nervous system), and the organism. In our study design and analysis, we control for many of these confounders. First, we excluded subjects with neurological or psychiatric diseases (or first degree relatives of psychiatric patients), as well as of those with deleterious habits, such as alcoholism or substance abuse, given their relationship with religiosity [69], [70] and their effect on regional grey matter [71], [72]. Second, we controlled for the effects of the important *biological* confounders of age, gender and total intracranial volume (a surrogate for genetic and environmental factors influencing global brain development) by including them as covariates in the model. Third, we included as covariate the important *social* confounder of education (which presumably induces widespread brain reorganization). Fourth (and perhaps most importantly), we calculated the global mean for grey matter for each subject, as a surrogate for *any* confounder's global effect on grey matter volume, and performed global normalization using an Analysis of Variance approach. This way we identified regions where the trends in grey matter volume differ from the total grey matter volume [67]. Admittedly, we did not correct for the potential confounding effect of *general health* (including vascular disease, diabetes etc). Nevertheless, we do not believe that their effect raises serious doubts over the validity of the findings: associations between general health and religiosity exist, but are generally indirect [73], [74] and there is no reason to suspect that their effect in regards to cortical volume is regionally specific.

## Supporting Information

Table S1 Participants' religiosity survey
(0.05 MB DOC)
