# Supplementary material for: Neuroanatomical Variability of Religiosity
Source: PLoS One. 2009 Sep 28;4(9):e7180. doi: 10.1371/journal.pone.0007180 (PMC2746321; doi:10.1371/journal.pone.0007180)
Supplement: Table S1 — Participants' religiosity survey (0.05 MB DOC) [file pone.0007180.s001.doc]

| ***Rate each of these items on a scale 1-7, where 1 = “I do not have this characteristic at all” and 7 = “I have this characteristic to a large extent”*** |
| --- |
| What is the degree of your current religiosity? |
| What is the degree of your current religious participation? |
| How often do you currently pray? |
| How often do you currently pray in private? |
| How often do you currently read the Scripture? |
| To what extent does religion influence important decisions in your life? |
| To what extent do you believe in Life after Death? |
| To what extent do you believe in Heaven? |
| To what extent do you believe in Hell? |
| To what extent do you believe in a personal God? |
| To what extent do you perceive God’s Love? |
| How often do you currently pray for forgiveness of your sins? |
| To what extent do you fear God’s anger or that God will punish you? |
| To what extent do you seek God’s will? |
| To what extent do you perceive God as being close to you? |
| To what extent do you have an awareness of God’s presence in your life? |
| To what extent do you perceive God as a friend? |
| To what extent do you perceive God’s fellowship? |
| To what extent do you rely on God for important decisions in your life? |
| To what extent do you doubt that God really exists? |
| What was the degree of your religiosity during upbringing? |
| What was the degree of your religious participation during upbringing? |
| How often were you praying during upbringing? |
| To what extent do you believe in “Eat, drink and be merry, because tomorrow we die”? |
| To what extent do you believe that humanity is fundamentally equally good and bad (or neither good nor bad)? |
| To what extent do you believe that values are relative depending on the situation? |
| To what extent do you believe that life has an ultimate purpose? |
